# Remote Ischemic Conditioning in the Prevention for Stroke-Associated Pneumonia: A Pilot Randomized Controlled Trial

**DOI:** 10.3389/fneur.2021.723342

**Published:** 2022-02-03

**Authors:** Bowei Zhang, Wenbo Zhao, Hongrui Ma, Yunzhou Zhang, Ruiwen Che, Tingting Bian, Heli Yan, Jiali Xu, Lin Wang, Wantong Yu, Jia Liu, Haiqing Song, Jiangang Duan, Hong Chang, Qingfeng Ma, Qian Zhang, Xunming Ji

**Affiliations:** ^1^Department of Neurology, Xuanwu Hospital of Capital Medical University, Beijing, China; ^2^Department of Neurology, Beijing Fengtai You'anmen Hospital, Beijing, China; ^3^Department of Hematology, Xuanwu Hospital, Capital Medical University, Beijing, China; ^4^China-America Institute of Neuroscience, Xuanwu Hospital, Capital Medical University, Beijing, China; ^5^Department of Emergency, Xuanwu Hospital of Capital Medical University, Beijing, China; ^6^Department of Neurosurgery, Xuanwu Hospital of Capital Medical University, Beijing, China

**Keywords:** stroke, stroke-associated pneumonia (SAP), remote ischemic conditioning (RIC), stroke-induced immunodepression (SIID), immune response

## Abstract

**Background:**

Despite the continuing effort in investigating the preventive therapies for stroke-associated pneumonia (SAP), which is closely associated with unfavorable outcomes, conclusively effective therapy for the prevention of SAP is still lacking. Remote ischemic conditioning (RIC) has been proven to improve the survival in the sepsis model and inflammatory responses have been indicated as important mechanisms involved in the multi-organ protection effect of RIC. This study aimed to assess the safety and the preliminary efficacy of RIC in the prevention of SAP in patients with acute ischemic stroke.

**Methods:**

We performed a proof-of-concept, pilot open-label randomized controlled trial. Eligible patients (age > 18 years) within 48 h after stroke onset between March 2019 and October 2019 with acute ischemic stroke were randomly allocated (1:1) to the RIC group and the control group. All participants received standard medical therapy. Patients in the RIC group underwent RIC twice daily for 6 consecutive days. The safety outcome included any adverse events associated with RIC procedures. The efficacy outcome included the incidence of SAP, changes of immunological profiles including mHLA-DR, TLR-2, and TLR-4 as well as other plasma parameters from routine blood tests.

**Results:**

In total, 46 patients aged 63.1 ± 12.5 years, were recruited (23 in each group). Overall, 19 patients in the RIC group and 22 patients in the control group completed this study. No severe adverse event was attributed to RIC procedures. The incidence of SAP was lower in the remote ischemic conditioning group (2 patients [10.5%]) than that in the control group (6 patients [27.3%]), but no significant difference was detected in both univariate and multivariate analysis (*p* = 0.249 and adjusted *p* = 0.666). No significance has been found in this pilot trial in the level of immunological profiles HLA-DR, TLR4 and TLR2 expressed on monocytes as well as blood parameters tested through routine blood tests between the two groups (*p* > 0.05). The IL-6 and IL-1β levels at day 5 after admission in the RIC group were lower than those in the control group (*p* < 0.05).

**Interpretation:**

This proof-of-concept pilot randomized controlled trial was to investigate RIC as a prevention method for SAP. Remote ischemic conditioning is safe in the prevention of SAP in patients with acute ischemic stroke. The preventive effect of RIC on SAP should be further validated in future studies.

## Introduction

Stroke-associated pneumonia refers to a spectrum of pulmonary infections in patients within 7 days after stroke onset (1). Stroke-associated pneumonia (SAP) is one of the most common complications after stroke, which has been well-known for worsening patient outcomes including the increasing disability and mortality with an estimated incidence ranging from 5–26% in general (2–5). Even though continuing effort in investigating the preventive therapies for SAP especially preventive antibiotics, conclusively effective therapy for the prevention of SAP is still lacking (6, 7).

Remote ischemic conditioning (RIC) is a strategy to protect distant organs against lethal acute ischemia-reperfusion injury which can be applied ≥ 1 cycle of brief and non-lethal limb ischemia through simply inflating and deflating a standard blood-pressure cuff placed on the upper arm (5, 8–10). Evidence shows that the survival has been prolonged in the sepsis model of rodents and sheep through applying the RIC procedure (11–13). Even though the mechanisms of the protective effect of RIC are not completely understood, the inflammatory responses have been indicated as important mechanisms involved in the multi-organ protection effect of RIC including the heart and brain (14, 15). However, whether RIC has a beneficial effect on SAP prevention is still not known. In this study, we aimed to assess the safety and the preliminary efficacy of RIC in the prevention of SAP in patients with acute ischemic stroke.

Clinical studies have shown that monocyte human leukocyte antigen-DR (mHLA-DR) and some blood parameters of the routine blood tests are associated with SAP occurrence (16–20). The loss of expression of mHLA-DR is suggested to be closely relevant to stroke-induced immunodepression which predisposes SAP (17–19). Monocytes are of vital significance in acute ischemic stroke (17, 21). In addition, toll-like receptors (TLRs) are pattern recognition receptors of innate immune cells. Inflammatory cells especially macrophages interact with damage-associated molecular patterns (DAMPs) released from destructed neural tissue from a stroke via TLRs, which can activate downstream inflammatory signaling pathways, play a critical role in the pathogenesis of stroke (21). Among TLR families, TLR2 and TLR4 are most studied which are closely relevant to the stroke-induced inflammatory response (17, 21–24). In this study, we examined HLA-DR, TLR-2, and TLR-4 of monocytes as well as routine blood tests to help determine the effects of RIC on SAP prevention.

## Methods

### Trial Design

This is a proof-of-concept, pilot open-label, and assessor-blinded randomized controlled trial conducted at Xuanwu Hospital of Capital Medical University. Patients were randomly assigned in a 1:1 ratio to undergo either remote ischemic conditioning or no intervention (control) group. This study was approved by the Ethics Committee of Xuanwu Hospital of Capital Medical University. All subjects or their legally authorized representative provided informed consent before enrollment.

### Participants

Eligible patients for enrollment were adults (≥ 18 years of age), have confirmed diagnosis of acute ischemic stroke (AIS) with the onset of symptoms within 48 h at recruitment, have NIHSS score ≤ 15, have pre-stroke modified Rankin Scale (mRS) ≤ 2, and subjects or their legally authorized representative was able to provide informed consent.

Exclusion criteria were uncontrolled hypertension (defined as systolic blood pressure ≥ 200 mmHg), participation in another device or drug trial simultaneously, any vascular, soft tissue, or orthopedic injury (e.g., superficial wounds and fractures of the arm) that contraindicated unilateral arm ischemic conditioning, peripheral vascular disease (especially subclavian arterial and upper limb artery stenosis or occlusion), stroke patients who underwent endovascular therapy for this time of onset, pregnancy, history of malignancies, using remote ischemic conditioning within the preceding 1 week, known dysphagia/nasogastric feeding tubes or infection at admission, a history of infection or the use of antibiotics, immunosuppressants, or steroids within the preceding 3 months, other conditions are not suitable for this trial (evaluated by researchers). Participants underwent follow-up assessment at 3-months through telephone or clinical visits.

### Interventions

Standard stroke medical therapy was consistent in all the enrolled patients in both the RIC group and the control group, including antiplatelet, statins, and risk factors management. The antihypertensive and antidiabetic agents were decided by the treating physicians who worked in the stroke unit based on the condition of the patients. Eligible patients allocated in the RIC group received RIC procedures twice daily for 6 consecutive days and the patients in the control group received standard stroke medical therapy only.

The RIC procedure consisted of five cycles of inflation to a pressure of 200 mmHg and deflation for 5 min alternately within 2 h of enrollment performed unilateral upper arm (the transfusion side was avoided) by an electric auto-controlled device with cuff (patent number ZL201420846209.5, China). This procedure can be stopped at any time if the subject experiences discomfort. The RIC procedure was done with the assistance of the nurses in the stroke unit.

### Randomization

All subjects were consecutively enrolled and randomly allocated in a 1:1 ratio to the RIC group (RIC procedure plus standard stroke medical therapy) or the control group (standard stroke medical therapy only) based on a computer-generated randomization code. The randomization of the assignment was sealed and handed to the treating physician who was blinded to the protocol.

### Outcome Assessment

#### Safety Outcome Assessment

The safety outcomes included the following: (1) inability to tolerate RIC procedure that leads to discontinuation; (2) objective signs of tissue or neurovascular injury resulting from RIC procedure; (3) stroke deterioration within 7 days (4); any hemorrhagic transformation within 7 days; (5) any adverse events within 90 days after stroke onset. The inspection was to evaluate especially for any local edema, erythema, skin lesions, and palpation of distal pulses of the upper arm, which was done by observers blinded to the study protocol. Any suspicious adverse event associated with the RIC procedure would be reported to the investigators.

#### Efficacy Outcomes Assessment

The efficacy outcome included: (1) the incidence of SAP; (2) Favorable outcome (modified Rankin Score (mRS) 0–2); (3) changes of immunological profiles including HLA-DR, TLR-2, and TLR-4 expressed on monocytes; (4) changes of the inflammatory cytokines including IL-1β, IL-6, TNFα, and IL-10. Based on the combination of clinical symptoms, radiological findings, and pathogen detection, SAP was diagnosed by the treating physician according to the modified Centers for Disease Control and Prevention criteria of stroke-associated pneumonia (1). In this study, the diagnosis of SAPs was reaching the definite SAP which means the diagnostic changes on the chest radiograph existed (Detailed diagnostic criteria can be seen in the Supplementary Material). Clinical outcome was evaluated according to the mRS. Favorable functional outcome was defined as mRS 0–2 at 90 days through telephone follow-up or clinical visits by certified vascular neurologists.

#### Flow Cytometry and Routine Blood Test

Baseline blood samples were collected between 5:00 a.m. and 6:30 a.m. after admission to the stroke care unit before any medication was started. Blood samples were collected at baseline, days 2 and 5 after admission.

Immunological profiles including the expression of HLA-DR, TLR-4, and TLR-2 on monocytes were measured through flow cytometry by investigators blinded to clinical endpoints. White blood cells were gated according to CD45+ expression. Monocytes were then gated according to CD14+ expression after removal of cell debris and non-leukocyte particles (Supplementary Material). Specifically, 2-ml of blood samples were collected in EDTA-coated tubes. 100 μl of anticoagulated whole blood was added to the mixture of a selected panel of monoclonal antibodies.

The following monoclonal antibodies were used: Fluorescein isothiocyanate (FITC) anti-human CD14 antibody, PerCP anti-human CD45 antibody, phycoerythrin (PE) anti-human CD284 (TLR4) antibody, Alexa Fluor647 anti-human CD282 (TLR2) antibody, allophycocyanin/cyanine7 (APC/Cy7) anti-human HLA-DR antibody (All from BioLegend, San Diego, CA, USA). FITC- mouse IgG2a k Isotype Control, Alexa Fluor647 mouse IgG2a k Isotype Control, and APC/Cy7 mouse IgG2a k Isotype Control were used as quality controls (BioLegend, San Diego, CA, USA). Monoclonal antibodies were mixed with the cell suspension and incubated at room temperature for 20 min in the dark. After erythrocyte lysis of the whole blood/antibody mixture and incubation at room temperature for 10 min. The acquisition was performed by an LSR II flow cytometer (BD Biosciences, San Diego, CA, USA) with Diva version 6.1.3 (BD Biosciences, San Diego, CA, USA), Flowjo software (BD Biosciences, San Diego, CA, USA), Sysmex XE-5000 autoanalyzer (MEK-7222K, Nihon Kohen, Japan), Meso Scale Discovery (MSD, Rockville, Maryland, USA) kit of the cell resuspension solution. Flowjo software was used for analysis.

Venous blood samples were collected into standardized tubes containing an anticoagulant (EDTA) and stored at room temperature. We measured white blood cell, neutrophil, lymphocyte, and monocyte counts through routine blood tests, which were determined using Sysmex XE-5000 autoanalyzer within 1 h after sample collection.

### Measurement of Inflammatory Cytokines

Plasma was obtained at baseline, day 2 (24 h after RIC/ no-RIC procedure), and day 5 after admission and stored at −80°C. The plasma inflammatory cytokines including the pro-inflammatory IL-1β, IL-6, and TNFα, as well as the anti-inflammatory IL-10, were measured through ELISA assay conducted with the Meso Scale Discovery (MSD, USA) kit following the manufacturer's instructions.

### Statistical Analysis

We compared the data between participants treated with and without RIC. The analysis was based on the per-protocol population, defined as randomly allocated individuals in their assigned group. Continuous variables were exhibited by mean ± *SD* for normal distribution or median while interquartile range (IQR) for skewed distribution. Independent *t*-test or Mann–Whitney *U*-test for continuous variables, and the Chi-Square test or Fisher's exact test for categorical variables were used to detect the differences between two groups. The normality of distributions was tested by the Kolmogorov-Smirnov test. A multiple logistic regression model was used to adjust the baseline characteristics between the two groups. All data were processed using SPSS 23 for Mac (IBM, Chicago, IL, USA) with a significance level of *P* < 0.05 (two-sided).

### Data Availability

Any data not published within the article will be shared in anonymized form by request from any qualified investigator.

## Results

**Figure 1:**
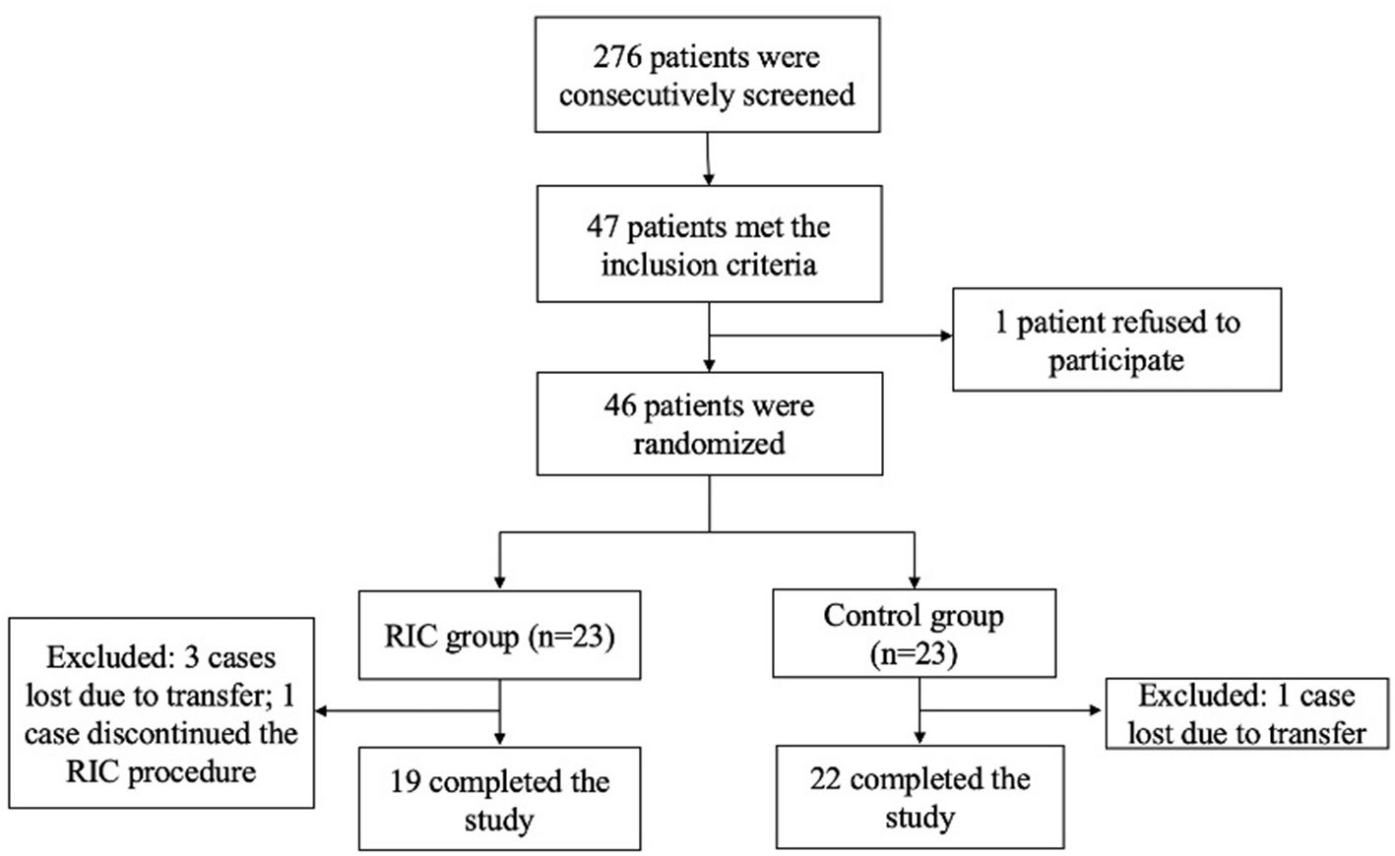
Enrollment and randomization. RIC, remote ischemic conditioning.

Between March 2019 and October 2019, 276 patients were consecutively screened in an advanced stroke center (Xuanwu Hospital of Capital Medical University). Forty seven patients met the inclusion criteria. One patient was reluctant to participate in this study. Forty six patients were enrolled and underwent randomization equally to the RIC group and the control group (23 in each group). 3 cases were lost in the RIC group due to remote transferring where the RIC facilities were not available. Ultimately, a total of 19 patients in the RIC group and 22 patients in the control group completed the observation during admission and 90 days follow-up (Figure 1).

### Baseline Characteristics

Baseline characteristics of 41 subjects were summarized in Table 1. The mean age was 63.1 ± 12.5 years old with a median National Institutes of Health Stroke Scale (NIHSS) score of 6 (4–10). Twenty six patients (63.4%) were men. The age, gender, baseline NIHSS score, and stroke etiology did not differ significantly among these 2 groups. The comorbidities were shown comparable between the RIC group and the control group except the rate of hypertension was higher in the control group (*p* = 0.008).

**Table 1:** Baseline characteristics between the RIC group and control group in patients with acute ischemic stroke.

	**All (*N =* 41)**	**RIC group (*N =* 19)**	**Control group (*N =* 22)**
Age, y (mean ± SD)	63.1 ± 12.5	61.6 ± 10.9	64.3 ± 13.6
Male, *n* (%)	26 (63.4)	11 (57.9)	15 (68.1)
Baseline NIHSS score, median (IQR)	6 (4–10)	6.5 (4.3–7.8)	6 (4–12)
Anterior circulation, *n* (%)	28 (68.3)	13 (68.4)	15 (68.2)
Posterior circulation, *n* (%)	13 (31.7)	6 (31.6)	7 (31.8)
**Comorbidities**, ***n*** **(%)**			
Hypertension	26 (63.4)	8 (42.1)	18 (81.8)
Diabetes Mellitus	18 (43.9)	9 (47.4)	9 (40.9)
Atrial fibrillation	4 (9.8)	1 (5.3)	3 (13.6)
Hyperlipidemia	13 (31.7)	5 (26.3)	8 (36.4)
Previous stroke history	9 (22.0)	5 (26.3)	4 (18.2)
Coronary artery disease	5 (12.2)	2 (10.5)	3 (13.6)
Smoking	16 (39.0)	7 (36.8)	9 (40.9)
**Stroke etiology**, ***n*** **(%)**			
Large-artery atherosclerosis	22 (53.7)	11 (57.9)	11 (50.0)
Cardioembolism	3 (7.3)	1 (5.3)	2 (9.1)
Small-vessel occlusion and other causes	16 (39.0)	7 (36.8)	9 (40.9)
**Treatment**, ***n*** **(%)**			
**IV tPA**	3 (7.3%)	2 (10.5%)	1 (4.5%)

*RIC, remote ischemic conditioning; SD, standard deviation; IQR, interquartile range; NIHSS, National Institutes of Health Stroke Scale; IV tPA, intravenous tissue plasminogen activator*.

### Safety Outcomes

No severe adverse event was attributed to RIC procedures. The RIC procedures were well tolerated. One patient in the RIC group experienced skin petechiae of the upper arm from repeated pressure cuff applications and decided to discontinue this procedure. 19 (82.6%) patients completed the RIC procedure overall and the per-protocol completion rate was 95%. No other signs of tissue or neurovascular injuries resulting from the RIC procedure including local edema, erythema, or skin lesions were observed. No subjects in both groups experienced hemorrhagic transformation. One patient in the control group showed stroke deterioration which led to death.

### Efficacy Outcomes

#### Clinical Events

Stroke-associated pneumonia (SAP) occurred in 8 (19.5%) of the patients. Two (10.5%) in the RIC group, 6 (27.3%) in the control group. The incidence of SAP was lower in the remote ischemic conditioning group (2 patients [10.5%]) than that in the control group (6 patients [27.3%]), but no significant difference was detected in both univariate (*p* = 0.249) and multivariate analysis (adjusted OR: 0.343, 95% CI: 0.043–2.758, *p* = 0.666) (Table 2).

**Table 2:** SAP incidence and favorable outcome at 3-month.

	**All (*N =* 41)**	**RIC group (*N =* 19), *n* (%)**	**Control group (*N =* 22), *n* (%)**	***P***
SAP	8 (19.5%)	2 (10.5%)	6 (27.3%)	0.249
mRS: 0–2	25 (61.0)	12 (63.2%)	13 (59.1%)	0.796

*RIC, remote ischemic conditioning*.

Further results showed that 25 patients (61%) [12 (63.2%) in the RIC group and 13 (59.1%) in the control group] demonstrated favorable outcomes. The favorable outcomes at 3-month between the two groups did not reach a statistical significance (*p* = 0.796) (Table 2).

#### Time Course of the Phenotype of Monocytes After Stroke

**Figure 2:**
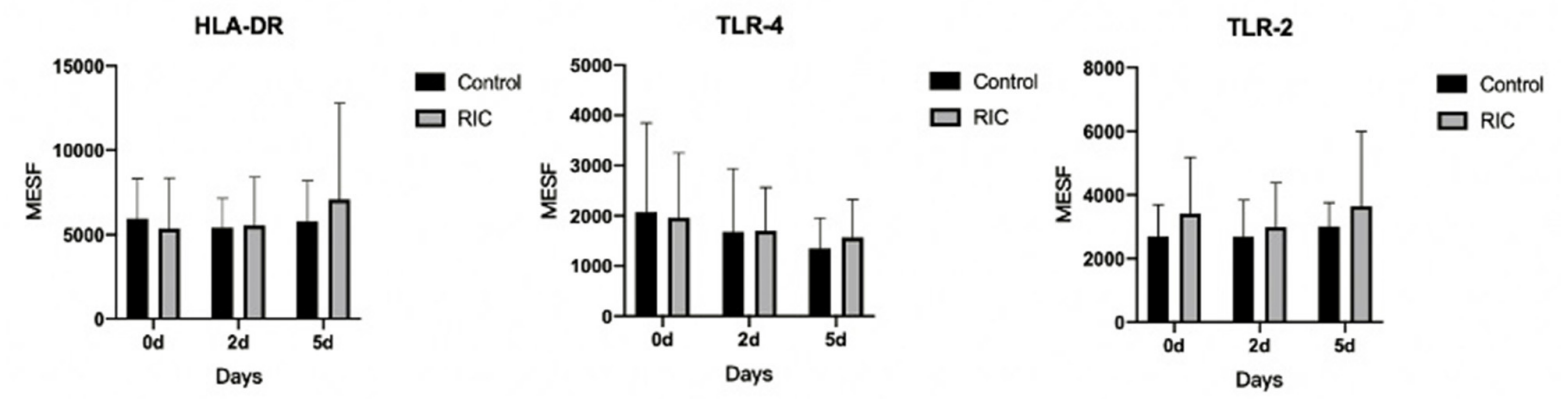
Time course after admission and phenotype of circulating monocytes in stroke patients between RIC group and the control group. RIC, remote ischemic conditioning; MESF, molecules of equivalent soluble fluorochrome.

The plasma expression of HLA-DR, TLR2, and TLR4 on monocytes was tested on the baseline, days 2 and 5. However, no statistical significance was detected in this pilot trial among these two groups (*p* > 0.05). No significance has been found in this pilot trial in the level of immunological profiles (Figure 2) as well as other plasma parameters between the two groups (Supplementary Material).

#### Time Course of Inflammatory Cytokines After Stroke

**Figure 3:**
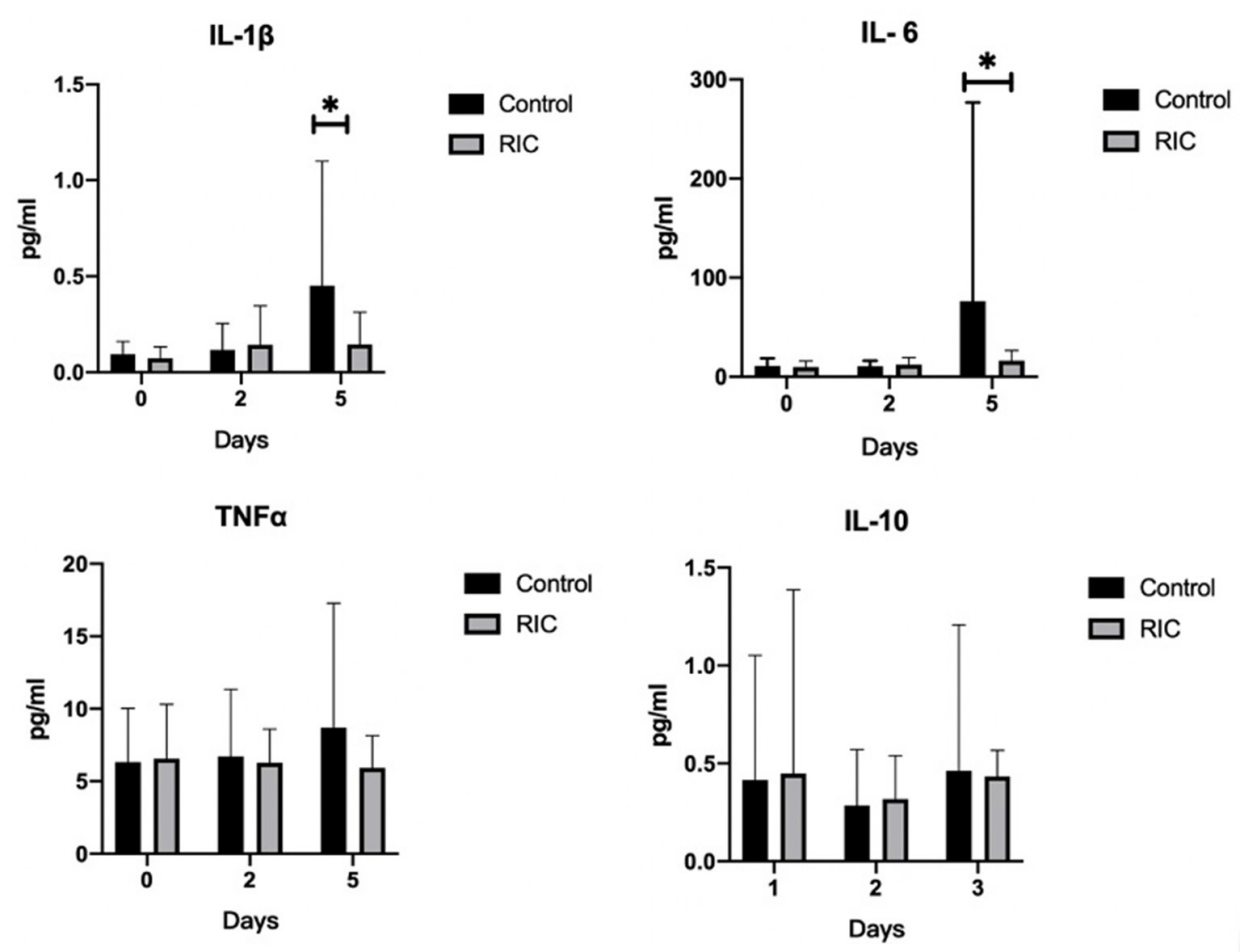
Pro-inflammatory and anti-inflammatory cytokines between RIC group and the control group. IL-1β, IL-6 and TNFα are as pro-inflammatory cytokines and IL-10 is as anti-inflammatory cytokine. Data are expressed as mean ± SD. ^*^P < 0.05. RIC, remote ischemic conditioning.

We measured the inflammatory cytokine including the pro-inflammatory IL-1β, IL-6, and TNFα, as well as the anti-inflammatory IL-10. There was no statistical significance between the RIC group and the control group at baseline and day 2 after admission (*P* > 0.05). However, the IL-6 and IL-1β levels at day 5 after admission were higher in the control group than those in the RIC group (*P* < 0.05), while the level of TNFα and IL-10 remained comparable at day 5 after admission (Figure 3).

## Discussion

This proof-of-concept pilot randomized controlled trial indicated that it was safe to perform RIC in patients with acute ischemic stroke. Even though the incidence of SAP was lower in the RIC group than that in the control group, no significant difference was detected in both univariate and multivariate analyses. RIC seems to be an applicable clinical adjuvant treatment for stroke patients. The efficacy of the preventive effect of RIC on reducing SAP should be further validated in future studies.

This study indicated that RIC was well tolerated by patients with acute ischemic stroke. Despite 1 patient in the RIC group experiencing skin petechiae of the upper arm from repeated pressure cuff applications which lead to the patient's decision the discontinuation of the RIC procedure. No severe local or systematic adverse event was associated with RIC procedures.

The immune responses interacted with the CNS have been indicated in numerous studies of stroke and inflammation has been considered as an important target for stroke therapy (21, 25, 26). Stroke-induced immunodepression caused by neural injury of stroke was widely acknowledged as the underlying mechanism of stroke-associated pneumonia (18, 21, 27). Moreover, monocyte as an important part of our innate immunity plays a significant role in stroke-induced infections, and decreased expression of monocytic HLA-DR has been considered as a predictor of stroke-induced pneumonia (17, 18, 28). An increased number of monocytes can be found in the blood in stroke patients shortly after stroke and the deactivation of antigen-presenting molecules is considered to be a risk factor of poststroke infections (17, 28). TLR4 and TLR2 are pattern recognition receptors that can be sensed by immune innate cells and activate downstream signaling inflammatory cascade, which are the most investigated receptors associated with stroke-induced inflammation response (21).

The mechanisms of the protection of the RIC procedure have not been well elucidated. However, numerous studies have indicated that the RIC exerts its protection effect has been largely associated with its anti-inflammatory effect, not just in neuroprotection, but in multiorgan as well, including changes in the expression of inflammatory genes, attenuating the pro-inflammatory cytokines, and downregulating the pro-inflammatory signaling pathway involving nuclear factor kappa B (NF-κB) (15, 29–33). The survival rates in the sepsis model of rodents and sheep have been shown to improve through applying the RIC procedure (11–13). However, few clinical studies have focused on the effect of RIC on inflammatory responses. In this pilot randomized trial, we particularly focused on the prevention effect of the RIC procedure on reducing SAP, which is a common complication after stroke which is closely relevant to unfavorable outcomes (3, 4).

The clinical outcome indicated that the RIC may have a potential protective effect on the prevention of SAP in patients with acute ischemic stroke. A limited sample size of this pilot trial may be one of the reasons explaining the statistical insignificance of this pilot clinical trial. In this study, we detected the pro-inflammatory cytokines IL-6 and IL-1βat day 5 after admission in the RIC group were significantly lower than those in the control group but not at day 2. This result was consistent with the animal studies that RIC can attenuate the pro-inflammatory cytokines (11, 13). It suggests that restoring the imbalance between pro-inflammatory and anti-inflammatory cytokines might be an underlying mechanism for RIC to exert its protective effect.

There are several limitations in this pilot trial. First, due to our limited study population and clinical heterogeneity in this exploratory pilot clinical trial, even though the incidence of SAP was lower in the RIC group than that in the control group, no significant difference was detected. Although statistical significances of IL-1βand IL-6 at day 5 after admission were detected between the two study groups, the result should be interpreted with caution and the protective effect of the RIC procedure on SAP should be further validated in future clinical trials in larger populations. Second, the RIC procedure in this study contained twice daily of 5 min ischemia and 5 min reperfusion for 5 cycles, which is a practical dose and may not be the optimal dose of RIC. Studies are still warranted to further investigate the optimal scheme of RIC in the clinical scenario of the intervention of acute ischemic stroke. Third, the effect of RIC on adaptive immunity was not evaluated in this study so that the specific underlying mechanisms of the protective effect of RIC should be further explored in future studies.

In conclusion, this proof-of-concept pilot randomized controlled trial was to investigate RIC as a prevention method for reducing SAP occurrence. Remote ischemic conditioning is safe in the prevention of SAP in patients with acute ischemic stroke. Attenuating the pro-inflammatory cytokines might be a way for RIC to exert its effect. However, the efficacy of RIC on the prevention of SAP and the underlying mechanisms should be further investigated in future studies.

## Data Availability Statement

The original contributions presented in the study are included in the article/Supplementary Material, further inquiries can be directed to the corresponding author.

## Ethics Statement

The studies involving human participants were reviewed and approved by The Ethics Committee of Xuanwu Hospital of Capital Medical University. The patients/participants provided their written informed consent to participate in this study.

## Author Contributions

BZ designed the study, collected, and analyzed the data, and drafted the manuscript. WZ and RC participated in the design of the study. HM, YZ, TB, HY, JX, LW, WY, JL, HS, JD, HC, QM, and QZ participated in the coordination of the study. XJ is the corresponding author and participated in the design and coordination of this study. All authors read and approved the final manuscript.

## Funding

This study was funded by The National Natural Science Foundation of China (No. 81771260), Chang Jiang Scholars Program (No. T2014251).

## Conflict of Interest

XJ is one of the inventors of the electronic auto-control device that has been patented in China (ZL201420846209.5, China). The remaining authors declare that the research was conducted in the absence of any commercial or financial relationships that could be construed as a potential conflict of interest.

## Publisher's Note

All claims expressed in this article are solely those of the authors and do not necessarily represent those of their affiliated organizations, or those of the publisher, the editors and the reviewers. Any product that may be evaluated in this article, or claim that may be made by its manufacturer, is not guaranteed or endorsed by the publisher.
