# Supplementary material for: Remote Ischemic Conditioning in the Prevention for Stroke-Associated Pneumonia: A Pilot Randomized Controlled Trial
Source: Front Neurol. 2022 Feb 3;12:723342. doi: 10.3389/fneur.2021.723342 (PMC8850400; doi:10.3389/fneur.2021.723342)
Supplement: Supplementary file 1 [file Table_1.DOCX]

**Supplementary material**

1. **Diagnostic criteria of SAP- Recommended diagnostic criteria for SAP not receiving mechanical ventilation based on CDC criteria^1^**

At least 1 among: Fever (>38°C) with no other recognized cause/ Leukopenia (<4000 WBC/mm^3^) or leukocytosis (>12 000 WBC/mm^3^)/ For adults ≥70 y old, altered mental status with no other recognized cause; And at least 2 among: New onset of purulent sputum, or change in character of sputum over a 24 h period, or increased respiratory secretions, or increased suctioning requirements/ New onset or worsening cough, or dyspnea, or tachypnea (respiratory rate>25/min) / Rales, crackles, or bronchial breath sounds/ Worsening gas exchange.

1. **Flow cytometry and routine blood tests**

**Table 1 HLA-DR expressed on monocytes**

| **HLA-DR**  **(MESF)**  **, median (IQR)** | **All**  **(N=41)** | **RIC group**  **(N=19)** | **Control group (N=22)** | **P** |
| --- | --- | --- | --- | --- |
| Baseline | 5243（3926-6523） | 4428（3349-6674） | 6102（4469-6484） | 0.297 |
| 2d | 5291（3715-6617） | 4500（3460-6988） | 5484（4367-6457） | 0.682 |
| 5d | 5553 (4458-7532) | 5553 (4477-7847) | 5309 (4453-6487) | 0.852 |

*HLA, human leukocyte antigen; MESF, molecules of equivalent soluble fluorochrome.*

**Table 2 TLR2 expressed on monocytes**

| **TLR2 (MESF)**  **, median (IQR)** | **All**  **(N=41)** | **RIC group**  **(N=19)** | **Control group (N=22)** | **P** |
| --- | --- | --- | --- | --- |
| Baseline | 2822（2229-3339） | 3146 (2330-3907) | 2628 (2229-3196) | 0.310 |
| 2d | 2700（1827-3632） | 2741 (1920-3632) | 2629 (1690-3512) | 0.501 |
| 5d | 2986 (2227-3914) | 3188 (1991-4012) | 2850 (2427-3632) | 0.901 |

*TLR，Toll-like receptors；MESF, molecules of equivalent soluble fluorochrome.*

**Table3 TLR4 expressed on monocytes**

| **TLR4 (MESF)**  **, median (IQR)** | **All**  **(N=41)** | **RIC group**  **(N=19)** | **Control group (N=22)** | **P** |
| --- | --- | --- | --- | --- |
| Baseline | 1652 (1095-1991) | 1450 (1088-1943) | 1707 (1176-2068) | 0.946 |
| 2d | 1266 (1013-2309) | 1592 (1040-2309) | 1127 (1002-1813) | 0.559 |
| 5d | 1396 (1016-1827) | 1484 (1120-1658) | 1372 (927-1894) | 0.495 |

*TLR，Toll-like receptors；MESF, molecules of equivalent soluble fluorochrome.*

**Table4 WBC count in RBT**

| WBC  ×10^3^/μL  , median (IQR) | All  (N=41) | RIC group (N=19) | Control group (N=22) | P |
| --- | --- | --- | --- | --- |
| Baseline | 7.96（6.74-9.38） | 7.45 (6.74-9.63） | 7.9 (6.9-8.7) | 0.340 |
| 2d | 7.85（6.87-9.58） | 7.22 (6.70-8.38) | 8.0 (7.4-9.3) | 0.118 |
| 5d | 7.74（6.89-10.59） | 7.47 (7.04-9.26) | 7.7 (6.7-9.5) | 0.507 |

*WBC, white blood cell; RBT, routine blood test; IQR, interquartile range*

**Table5 Neutrophils count in RBT**

| **Neutrophils**  **×10^3^/μL [IQR]** | **All (N=41)** | **RIC group (N=19)** | **Control group (N=22)** | **P** |
| --- | --- | --- | --- | --- |
| Baseline | 5.74 (4.28-7.36) | 5.08 (4.34-7.46) | 6.00 (4.28-7.28) | 0.316 |
| 2d | 5.36 (4.18-6.24) | 4.95 (4.07-6.15) | 5.46 (4.34-6.19) | 0.232 |
| 5d | 4.92 (4.37-7.42) | 4.92 (4.32-6.46) | 4.81 (4.46-6.54) | 0.644 |

*RBT, routine blood test; IQR, interquartile range*

**Table6 Lymphocytes count in RBT**

| **Lymphocytes**  **×10^3^/μL [IQR]** | **All (N=41)** | **RIC group (N=19)** | **Control group (N=22)** | **P** |
| --- | --- | --- | --- | --- |
| Baseline | 1.73 (1.24-2.23) | 1.91 (1.21-2.28) | 1.73 (1.24-2.12) | 0.750 |
| 2d | 2.01 (1.47-2.15) | 2.09 (1.36-2.35) | 1.97 (1.34-2.09) | 0.842 |
| 5d | 2.02 (1.48-2.32) | 2.11 (1.70-2.33) | 2.07 (1.39-2.43) | 0.795 |

*RBT, routine blood test; IQR, interquartile range*

**Table7 Monocytes count in RBT**

| **Monocytes**  **×10^3^/μL [IQR]** | **All (N=41)** | **RIC group (N=19)** | **Control group (N=22)** | **P** |
| --- | --- | --- | --- | --- |
| Baseline | 0.40 (0.33-0.46) | 0.41 (0.37-0.46) | 0.40 (0.33-0.48) | 0.762 |
| 2d | 0.41 (0.32-0.53) | 0.42 (0.37-0.44) | 0.43 (0.33-0.52) | 0.724 |
| 5d | 0.40 (0.34-0.54) | 0.39 (0.35-0.47) | 0.40 (0.33-0.55) | 0.773 |

*RBT, routine blood test; IQR, interquartile range*

1. **Figure 1 Flow cytometry analysis**


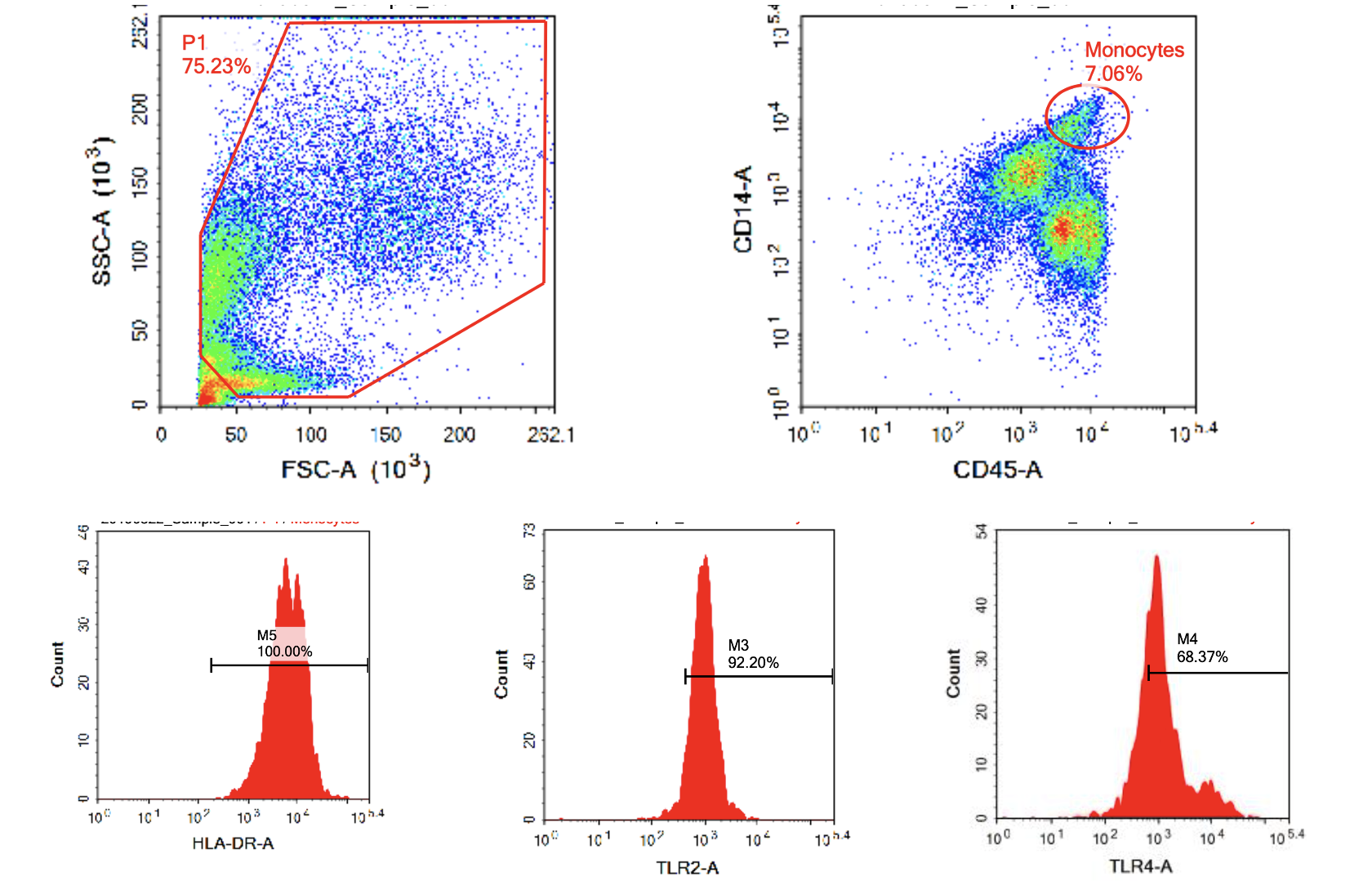


1. Smith CJ, Kishore AK, Vail A, Chamorro A, Garau J, Hopkins SJ, Di Napoli M, Kalra L, Langhorne P, Montaner J *et al*: **Diagnosis of Stroke-Associated Pneumonia: Recommendations From the Pneumonia in Stroke Consensus Group**. *Stroke* 2015, **46**(8):2335-2340.
